# Injustice Squared? An Intersectional Lens to Research on Productive Engagement in Later Life

**DOI:** 10.1093/geroni/igab046.083

**Published:** 2021-12-17

**Authors:** Christina Matz, Cal Halvorsen, Jacquelyn James

## Abstract

Social inequalities over the life course shape later life opportunities and outcomes in important ways. However, research on paid and unpaid work in later life has not always captured (and has sometimes mischaracterized) the variety and complexity of lived experiences in later life—in particular for low-income workers, workers of color, women, and others marginalized due to their social position. Further, statistics often obscure the most important information: how the most marginalized older workers are faring. Intersectionality, a term coined by legal scholar, Dr. Kimberlé Crenshaw, describes the overlapping and intersecting social identities that often influence how we move around in society. Some identities garner privilege and power and others oppression and marginalization; we must look at their intersection to better understand complexity and inform solutions. This symposium will apply an intersectional lens to research on paid and unpaid work in later life. The first paper is a scoping review that assesses the extent to which race and ethnicity are investigated in studies of the longitudinal association between workplace demands and cognitive health. The second paper explores how older Black and Hispanic adults’ work engagement is impacted by COVID-19. The third paper considers gender differences in volunteer engagement among Asian-American older adults. The final paper examines the Senior Community Service Employment Program’s role in participant financial, physical, and mental well-being. A discussant will reflect on these studies and the need for continued research that considers intersectionality in opportunities and experiences for paid and unpaid work in later life.

